# Hsa_circ_0128846 knockdown attenuates the progression of pancreatic cancer by targeting miR-1270/NR3C1 axis

**DOI:** 10.1038/s41598-023-28439-w

**Published:** 2023-02-16

**Authors:** Ming Wang, Ming Li, Zehan Liu, Cuinan Jiang, Hailong Lv, Qin Yang

**Affiliations:** Section for Hepatopancreatobiliary Surgery, Department of General Surgery, The Third People’s Hospital of Chengdu & The Affiliated Hospital of Southwest Jiaotong University, No. 19 Yangshi Road, Chengdu, 610031 Sichuan China

**Keywords:** Cell biology, Molecular biology

## Abstract

The considerable role of circular RNAs (circRNAs) make them prospective biomarkers in cancer therapy. Our study aimed to unveil the function of circ_0128846 in pancreatic cancer (PC). The expressions of circ_0128846, miR-1270 and NR3C1 mRNA were measured via RT-qPCR. The expressions of NR3C1 protein and apoptosis-related markers (Bax and Bcl-2) were measured via western blotting. CCK-8, colony-forming, or wound healing assay was respectively utilized to identify cell proliferation, growth and migration. Xenograft model was developed to evaluate tumor growth affected by circ_0128846 in vivo. The putative binding between miR-1270 and circ_0128846 or NR3C1 was testified by dual-luciferase reporter, RIP or pull-down assay. Circ_0128846 showed elevated expression in PC. Circ_0128846 deficiency restrained cancer cell proliferation, colony formation and migratory ability, enhanced cell apoptotic rate, and also impeded tumor development in vivo. Circ_0128846 directly targeted miR-1270 whose expression was declined in PC. The suppressive effects of silencing circ_0128846 on PC cell malignant phenotypes were largely reversed by miR-1270 inhibition. NR3C1 was targeted by miR-1270 and was highly regulated in PC. The repressive effects of NR3C1 knockdown on PC cell malignant phenotypes were partly abolished by miR-1270 inhibition. Circ_0128846 deficiency blocked PC progression via mediating the miR-1270/NR3C1 pathway, which partly illustrated PC pathogenesis.

## Introduction

Pancreatic cancer (PC) is one of the poorer prognoses among all solid tumors due to its high aggressiveness and low survival rate, with an overall five-year survival rate of approximately 10%^1,2^. Risk factors for PC contain individual characteristics, lifestyle and environment, and disease status^1^. Globally, the incidence of PC is expected to increase to 18.6/100,000 by 2050, with an average annual growth rate of 1.1%, implying that PC will pose a significant public health burden^3^. Studies manifest that early diagnosis is essential to improve patients’ prognosis and therapeutic outcomes^4^, which requires the identification of additional biomarkers closely associated with PC progression.

Circular RNAs (circRNAs) are newly characterized non-coding RNAs that have been proposed to be prospective biomarkers in cancer management^5^. Benefiting from their closed-loop structures, circRNAs show higher stability in comparison to linear RNA molecules. In addition, circRNAs can be monitored in multiple body fluids, such as blood, urine and saliva^6–8^. These characteristics make circRNAs ideal biomarkers. Growing evidence has proved that circRNAs dysregulated in cancer samples present crucial regulatory roles in cancer biological processes, including cancer cell growth, migration and tumorigenesis^8^. The implications of numerous circRNAs in PC development have attracted much public concern. For example, circ_0000069 was overexpressed in PC, and its depletion provoked cancer cell apoptosis and cell cycle arrest and thus blocked PC tumorigenesis^9^. CircRNA expression profile records a variety of circRNAs that are differently expressed in pancreatic ductal adenocarcinoma (PDAC), such as GSE69362 dataset. We analyzed this dataset and discovered that circ_0128846 expression was greatly elevated in PDAC. Previous studies have evidenced the upregulation of circ_0128846 in colorectal cancer and highlighted its considerable carcinogenic effects^10^. However, the detailed roles of circ_0128846 in PC have not been elucidated.

CircRNAs are canonical to act as competing endogenous RNAs (ceRNAs) and thus control the miRNA/mRNA networks to regulate carcinogenesis^11^. We performed bioinformatics assay to investigate the downstream miRNA/mRNA networks targeted by circ_0128846. MiR-1270 was computationally predicted as a target of circ_0128846 and previously recorded to repress the malignant progression of glioblastoma^12^, breast cancer^13^, and bladder cancer^14^. Whereas, the interplay between circ_0128846 and miR-1270 in PC development is not confirmed. Additionally, miR-1270 was calculated to contain binding site with NR3C1 3’UTR by bioinformatics analysis, hinting miR-1270 might bind to nuclear receptor subfamily 3 group C member 1 (NR3C1) 3’UTR to suppress NR3C1 expression. NR3C1 can encode the glucocorticoid receptor, thereby participating in cell growth, glucocorticoid-induced apoptosis, inflammation, and differentiation^15,16^. Increasing studies confirm that NR3C1 as an oncogenic factor has been widely testified in various cancers, such as breast cancer, colorectal cancer, acute myeloid leukemia^17–20^. However, NR3C1 targeted by miR-19b was reported to be downregulated in colon cancer, and NR3C1 overexpression relieved oxaliplatin chemoresistance by regulating PI3K/AKT/mTOR pathway^16^. Nonetheless, the details functional effects of NR3C1 in PC have not been addressed, and its binding with miR-1270 has not been confirmed.

In the current work, we for the first time studied the detailed functions of circ_0128846 on PC cell behaviors and tumor growth. Besides, we proposed the miR-1270/NR3C1 network downstream of circ_0128846 to address the regulatory mechanism of circ_0128846 in PC. This work mainly aimed to unveil the role of circ_0128846 in PC development.

## Materials and methods

### Clinical specimens

Tumor specimens and paired normal specimens were excised from 37 patients diagnosed with PC in The Third People’s Hospital of Chengdu & The Affiliated Hospital of Southwest Jiaotong University. The specimens after excising from body were administered with liquid nitrogen and stored in − 80 °C freezers. Study design was permitted by The Third People’s Hospital of Chengdu & The Affiliated Hospital of Southwest Jiaotong University. The inclusion criteria were as follows: (1) The patients were pathologically confirmed to suffer from PC; (2) Enrolled patients had signed written informed consent; (3) The patients had never received previous anti-cancer therapies (such as radiotherapy and chemotherapy). The exclusion criteria were as follows: (1) The patients had history of pancreatic disease; (2) The patients had other malignant cancers or systemic diseases; (3) The patients were pregnant or lactating. The clinical characteristics of patients with PC were shown in Table 1.

**Table 1:** The clinical characteristics of 37 patients with PC.

Characteristics	Cases (n = 37)
Gender	
Male	22
Female	15
Age	
< 55	18
≥ 55	19
Tumor size (cm)	
< 3	17
≥ 3	20
TNM stage	
I–II	14
III–IV	23
Perineural invasion	
Absent	16
Present	21
Vascular invasion	
Absent	16
Present	21

### Cell culture and transfections

PC cell lines (CFPAC-1, Patu-8988t, PANC-1 and SW1990) and pancreatic non-cancer cell line (hTERT-HPNE) were acquired from BeNa (Beijing, China). CFPAC-1 cells were cultured in DMEM (BeNa) added with 10% FBS (BeNa) conditions, and other cell lines were all cultured in RPMI1640 (BeNa) added with 10% FBS conditions, in a 37 °C incubator supplemented with 5% CO_2_.

Two circ_0128846-specific small interference RNAs (si-circ-1 and si-circ-2), NR3C1-specific small interference RNA (si-NR3C1) and scrambled control (si-NC) were produced by Geneseed (Guangzhou, China). MiR-1270 mimic (mimic), mimic negative control (NC), miR-1270 inhibitor (inhibitor) and inhibitor-NC were bought from Ribobio (Guangzhou, China). Then, these oligonucleotides (50 nM) were transfected into the PC cells using Lipofectamine 3000 reagent (Invitrogen, USA). After transfection for 24 h, the expressions of circ_0128846, NR3C1 or miR-1270 were checked by RT-qPCR or western blotting to determine the transfection efficiency.

### RT-qPCR

Trizol reagent (Beyotime, Shanghai, China) was employed for RNA isolation. RNA samples were next administered with the cDNA synthesis kit (Transgene, Beijing, China) or miRNA First-Strand cDNA synthesis kit (Tiangen, Beijing, China) as the matched protocol suggested. Subsequently, cDNA was administered with SYBR Green Mixture (Transgene) to examine the expressions of circ_0128846, NR3C1 or miR-1270 with the use of matched PCR primers (Table 2). Here, GAPDH or U6 was included to normalize the expression of circ_0128846 and NR3C1 or miR-1270, respectively. Relative expression was processed by the 2^−ΔΔCT^ method.

**Table 2:** Real-time PCR primer sequences.

Gene name	Sequence
circ_0128846	Forward 5'-GACCTCTGTCAGCGAGTTCC-3' Reverse 5'-GCTACTGGAGCCTGATGGAC-3'
NR3C1	Forward 5'-GGAAGTGTCATCGAGGAGGAGA-3' Reverse 5'-TCGCTGCTTGGAGTCTGATTG-3'
miR-1270	Forward 5'-CTGGAGATATGGAAGAGCT-3' Reverse 5'- CAGTGCGTGTCGTGGAGT-3'
GAPDH	Forward 5'-ATGCCTCCTGCACCACCAACTGCTT-3' Reverse 5'-TGGCAGTGATGGCATGGACTGTGGT-3'
U6	Forward 5'-CTCGCTTCGGCAGCACA-3' Reverse 5'-AACGCTTCACGAATTTGCGT-3'

### CircRNA location assay

RNA samples derived from cytoplasmic component or nuclear component of SW1990 and PANC-1 cells were extracted using the PARIS Kit (Invitrogen) as the protocol mentioned. GAPDH was applied to be internal control in cytoplasm, and U6 was applied to be internal control in nucleus.

### RNase R assay

RNA isolated from SW1990 and PANC-1 cells was administered with RNase R (Geneseed) at 37 °C environment for 15 min. Next, total RNA was scrambled into cDNA and quantified by RT-qPCR.

### CCK-8 assay

The cultured cells were maintained at 96-well plates (2 × 10^3^ cells/well) for the indicated time, such as 0, 24, 48 or 72 h. Cells at different times were treated with CCK-8 reagent (Sigma-Aldrich, USA) for 2 h, with 10 μL in each well. The absorbance of cells in each well was examined by a microplate reader (Molecular Devices, Shanghai, China).

### Western blotting

RIPA reagent from Beyotime was adopted to extract total proteins. After quantifying by BCA kit (Beyotime), protein samples were loaded to 10% SDS-PAGE for band separation. The separated bands were transferred to PVDF membranes and then blocked with 5% skim milk at room temperature for 1 h. After that, the membranes containing protein bands were exposed to the primary antibodies overnight at 4 °C environment, such as anti-Bax (ab32503, 1/5000; Abcam, USA), anti-Bcl-2 (ab32124, 1/1000; Abcam), anti-NR3C1 (DF2399, 1/1000; Affbiotech, Suzhou, China) and anti-GAPDH (ab181602, 1/5000; Abcam). Next, these membranes were exposed to the secondary antibody (ab205718, 1/20,000; Abcam) at room temperature for 2 h. Eventually, the protein signaling was checked using the enhanced chemiluminescence (ECL) reagent (Beyotime).

### Colony formation assay

Cultured cells in 6-well plates (300 cells/well) were maintained at 37 °C conditions containing 5% CO_2_. Cell medium was renewed every 2 days, and cells were cultured for 10 days. Colonies were fixed with 4% paraformaldehyde, stained with 0.25% crystal violet, and clone formations were observed under a light microscope.

### Wound healing assay

Cells were seeded into 24-well plates (2 × 10^4^ cells/well) until 90% confluence. Then, an artificial wound on cell surface was created by using a sterile pipette tip. After washing with PBS, cells were cultured with serum-free culture medium to avoid the effect of cell proliferation. After 24-h incubation, the distance of wound healing (at 0 h and 24 h) was observed by light microscopy (Nikon, Japan).

### Xenograft model construction

Circ_0128846-specific short-hairpin-RNA (sh-circ) and matched negative control (sh-NC) were synthesized and assembled into lentivirus vector by Geneseed. Lentivirus particles carrying sh-circ or sh-NC were produced by Geneseed. Balb/c mice (female, 6-week-old) were bought from Vital River (Beijing, China). After a week of acclimatization, the poorly growing mice were eliminated. A total of 10 mice were adopted in this work and randomly assigned into two groups (n = 5 per group). Mice in different groups received subcutaneous injection of SW1990 cells (2 × 10^6^ cells per mouse) that were infected with lentivirus particles carrying sh-circ or sh-NC. During tumor formation and development, tumor volume (length × width^2^ × 0.5) was weekly measured. After 5 weeks of tumor development, all mice were euthanized by pentobarbital sodium. Tumor nodes were excised from mice for additional analyses. The procedures of animal use were approved by The Third People’s Hospital of Chengdu & The Affiliated Hospital of Southwest Jiaotong University. This animal experiment was conducted in accordance with standards for the Care and Use of Laboratory Animals.

### Dual-luciferase reporter experiment

The wild-type (WT, harboring miR-1270 binding site) and mutant-type (MUT, harboring the mutated miR-1270 binding site) reporter plasmids of circ_0128846 were respectively constructed into pmirGLO vector by Genepharma (Shanghai, China). MiR-1270 mimic (using mimic-NC as the control) was transfected with the WT or MUT plasmid of circ_0128846 into the experimental cells. As for NR3C1, the WT (containing miR-1270 binding site), MUT1 (containing the mutated miR-1270 binding site at position 1), MUT2 (containing the mutated miR-1270 binding site at position 2) and Co-MUT (containing the mutated miR-1270 binding site at position 1 and 2) reporter plasmids were constructed. MiR-1270 mimic (using mimic-NC as the control) was transfected with these reporter plasmids respectively into SW1990 and PANC-1 cells. After 48-h transfection, luciferase activity was examined using the Luciferase Reporter Assay System (Promega, USA).

### RIP experiment

By using Magna RIP Kit (Merckmillipore, USA), SW1990 and PANC-1 cells were exposed to lysis buffer. Magnetic Beads were pre-coated with anti-Ago2 antibody or anti-IgG antibody (control). Cell lysates were next cultured with antibody-coated beads. RNA complexes enriched by Ago2 or IgG were isolated from beads, followed by detection using RT-qPCR.

### Pull-down experiment

Biotin-labeled molecular probes of miR-1270 (Bio-mimic) or NC (Bio-NC) were obtained from Ribobio. SW1990 and PANC-1 cells were introduced with Bio-mimic or Bio-NC and then lysed by lysis buffer (Invitrogen). RNA complexes in cell lysates were captured by Streptavidin-Dyna beads (Invitrogen). The abundance of NR3C1 in RNA complexes was examined by RT-qPCR.

### Statistical analysis

Three independent experiments were included in this work. Data processing was conducted using GraphPad Prism 7 (GraphPad, USA), and data were finally shown as mean ± standard deviation. Pearson’s correlation analysis was employed for the linear analysis of two groups of data. For difference comparison in different groups, Student’s *t*-test or analysis of variance was adopted. The *P*-value < 0.05 indicated significant difference in statistics.

### Ethics approval

The present study was approved by the Ethics Committee of The Third People’s Hospital of Chengdu & The Affiliated Hospital of Southwest Jiaotong University (Chengdu, China). The processing of clinical tissue samples is in strict compliance with the ethical standards of the Declaration of Helsinki. All patients signed written informed consent. This animal experiment was conducted in accordance with the ARRIVE guidelines and was authorized by the Ethics Committee of The Third People’s Hospital of Chengdu & The Affiliated Hospital of Southwest Jiaotong University.

### Consent to participate

All patients signed written informed consent.

## Results

### Circ_0128846 expression was reinforced in tumor samples and cell lines of PC

**Figure 1:**
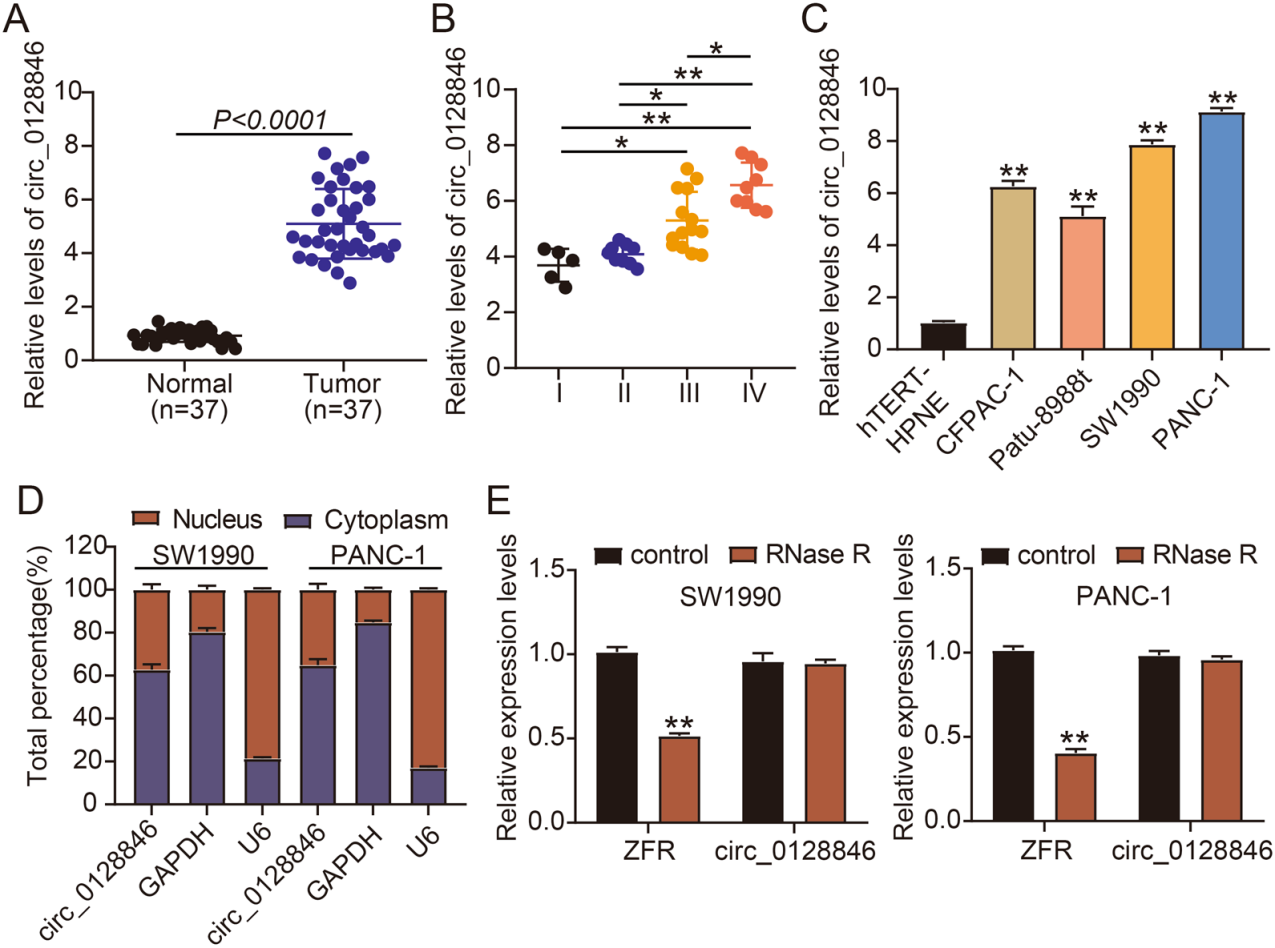
Circ_0128846 was highly expressed in PC. (**A**) Circ_0128846 expression in tumor samples of PC and normal samples was examined using RT-qPCR. (**B**) Circ_0128846 expression in different TNM stage. *P < 0.05, **P < 0.001. (**C**) Circ_0128846 expression in PC cell lines (CFPAC-1, Patu-8988t, PANC-1 and SW1990) and non-cancer cell line (hTERT-HPNE) was examined using RT-qPCR, **P < 0.001 relative to hTERT-HPNE. (**D**) The distribution of circ_0128846 in cytoplasm or nucleus of SW1990 and PANC-1 cells was ensured by RT-qPCR. (**E**) The reality of circ_0128846 was assessed using RNase R assay, **P < 0.001 relative to control.

To ascertain the expression level of circ_0128846 in PC, we performed RT-qPCR assay. Circ_0128846 expression was remarkably strengthened in PC specimens in contrast to normal specimens (Fig. 1A), and circ_0128846 expression in advanced tumor node metastasis (TNM) stage was upregulated (Fig. 1B). As expected, its expression was also highly elevated in PC cell lines (CFPAC-1, Patu-8988t, PANC-1 and SW1990) in contrast to non-cancer cell line (hTERT-HPNE) (Fig. 1C). Given that relatively high expression of circ_0128846 was observed in PANC-1 and SW1990 cells, these two cell lines were used in further experiments. Next, we concluded that circ_0128846 was prominently located in the cytoplasm section and slightly distributed in the nuclear section of PC cells (Fig. 1D). In SW1990 and PANC-1 cells-derived RNA samples with RNase R treatment, we noticed that circ_0128846 expression was rarely decreased, while its linear gene ZFR expression was greatly degraded (Fig. 1E), verifying the reality of circ_0128846. To sum up, circ_0128846 overexpression might be associated with PC progression.

### Silencing circ_0128846 repressed PC cell growth and migration but accelerated cell apoptosis

**Figure 2:**
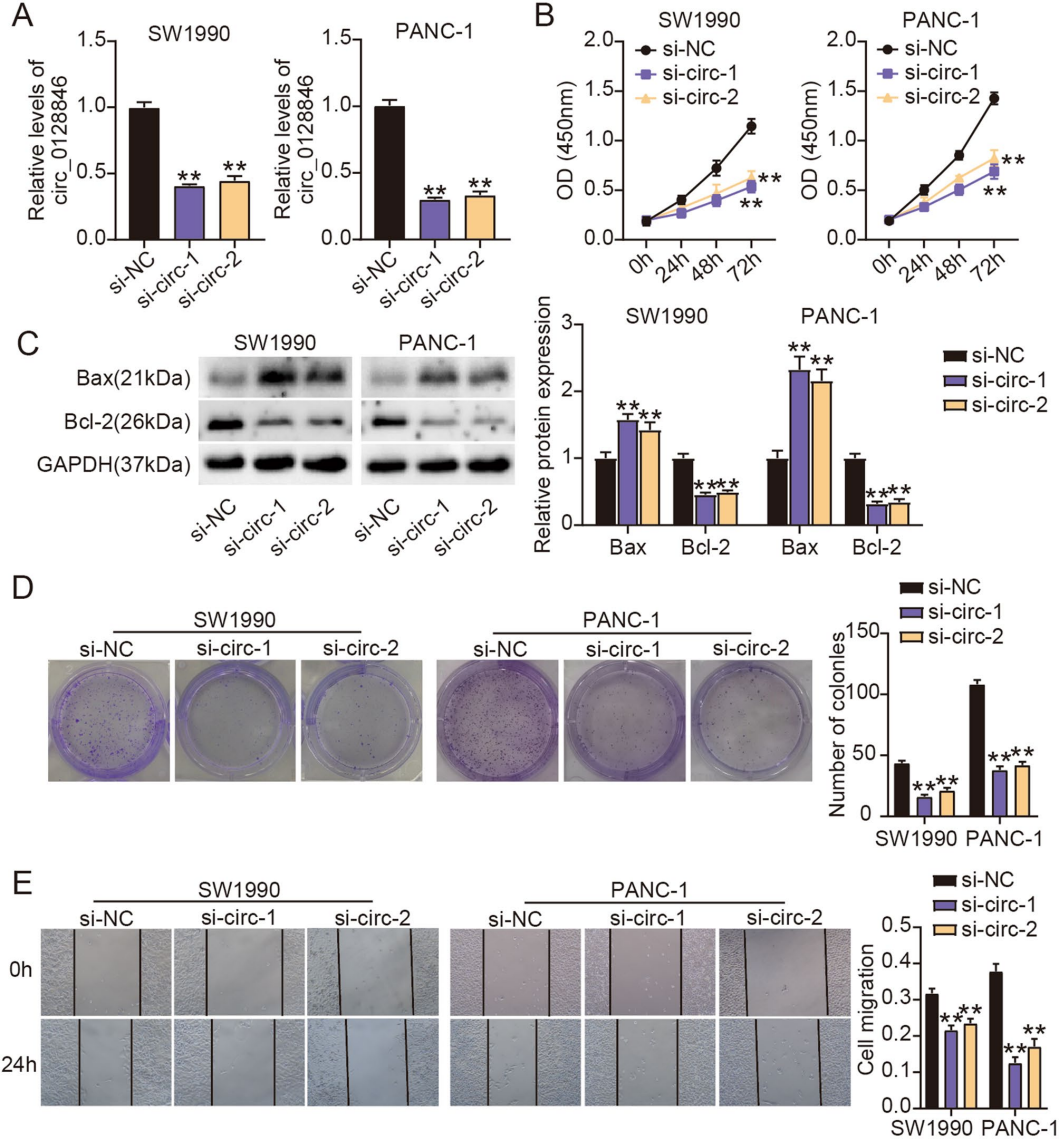
Silencing circ_0128846 restrained PC cell proliferation, colony formation and migration whereas enhanced cell apoptosis. (**A**) Circ_0128846 expression in SW1990 and PANC-1 cells transfected with two si-circ_0128846 (si-circ-1, si-circ-2) or si-NC was checked by RT-qPCR. (**B**) The effect of circ_0128846 silencing on cell proliferation was assessed by CCK-8 assay. (**C**) The expression of apoptosis-related proteins (Bax and Bcl-2) affected by circ_0128846 silencing was examined by western blotting. (**D**) The effect of circ_0128846 silencing on clone formation ability was assessed by colony formation assay. (**E**) The effect of circ_0128846 silencing on cell migration was assessed by wound healing assay. **P < 0.001 relative to si-NC.

We depleted circ_0128846 expression in PC cells to explore its functions. As a result, SW1990 and PANC-1 cells transfected with si-circ-1 and si-circ-2 showed the decreased expression of circ_0128846 (Fig. 2A). For function analysis, SW1990 and PANC-1 cells with silencing circ_0128846 had decreased proliferative ability by CCK-8 experiment (Fig. 2B). In addition, Bax level was markedly strengthened, while Bcl-2 level was markedly weakened in SW1990 and PANC-1 cells after circ_0128846 downregulation (Fig. 2C, Supplementary Information). The colony-forming ability of SW1990 and PANC-1 cells after circ_0128846 downregulation was largely declined (Fig. 2D). Furthermore, SW1990 and PANC-1 cells with si-circ transfection had repressive migratory capacity by wound healing experiment (Fig. 2E). Since the effect of si-circ-1 on PC cells was more significant, we used si-circ-1 to perform the following experiments. Overall, silencing circ_0128846 repressed PC cell malignant behaviors.

### Circ_0128846 absence hindered the development of in vivo tumors

**Figure 3:**
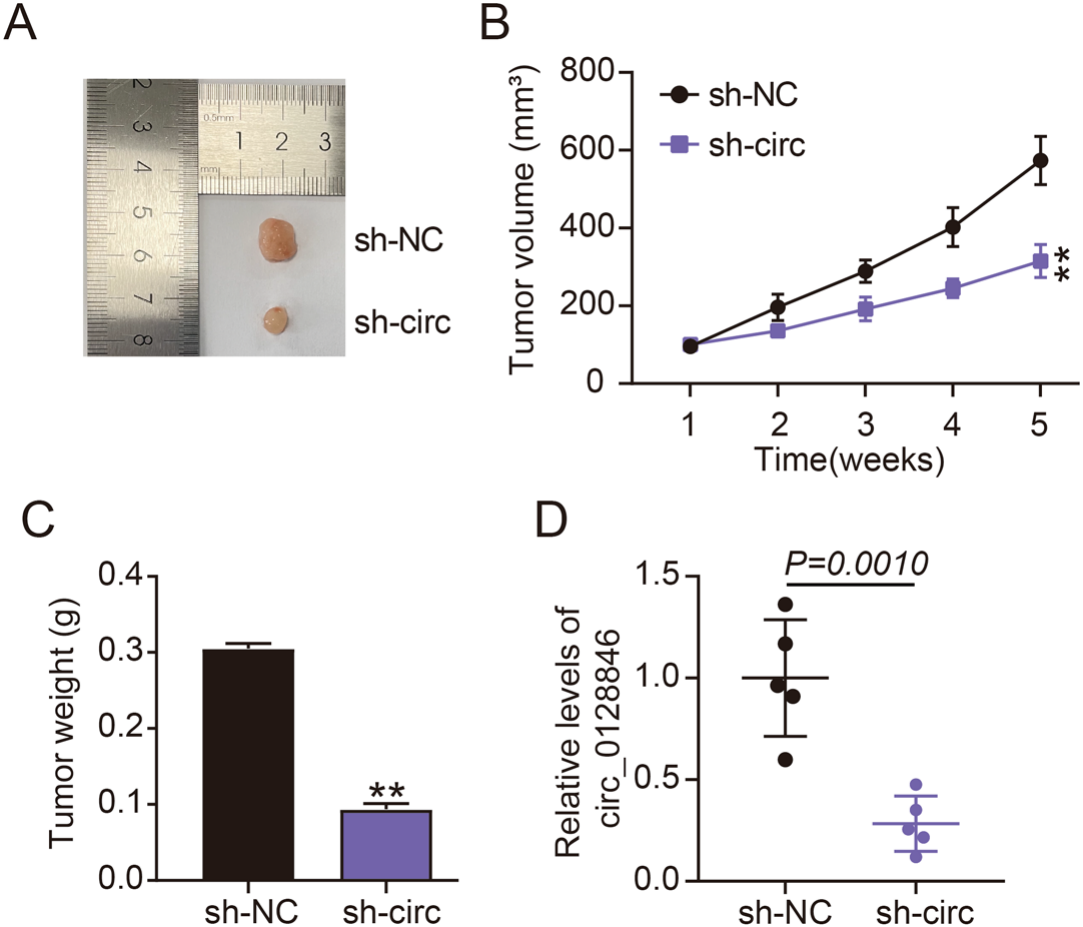
Circ_0128846 absence restrained tumor growth in vivo. (**A**–**C**) SW1990 cells infected with lentivirus of sh-circ or sh-NC were injected into nude mice to construct xenograft models. (**A**) Representative images of tumor tissues from sh-NC or sh-circ group. (**B**) Tumor volume was measured weekly to assess tumor growth. (**C**) After tumor growth for 5 weeks, tumor tissues were excised for weight measurement. (**D**) circ_0128846 expression in sh-NC and sh-circ group was detected by RT-qPCR. **P < 0.001 relative to sh-NC.

After realizing the role of circ_0128846 in cellular conditions, we constructed animal models to explore its role in vivo. As seen in Fig. 3A, the tumor size in mice that were administered with hypodermical injection of sh-circ lentivirus-infected SW1990 cells was much smaller than that in sh-NC-administered mice. In detail, tumor volume measured once a week was strikingly lower in the sh-circ group than that in sh-NC group (Fig. 3B). The weight of excised tumor tissues from sh-circ group was also much lower than that from sh-NC group (Fig. 3C). In addition, circ_0128846 expression in sh-circ group was reduced (Fig. 3D). Overall, circ_0128846 absence hindered the development of in vivo tumors.

### Circ_0128846 directly targeted miR-1270

**Figure 4:**
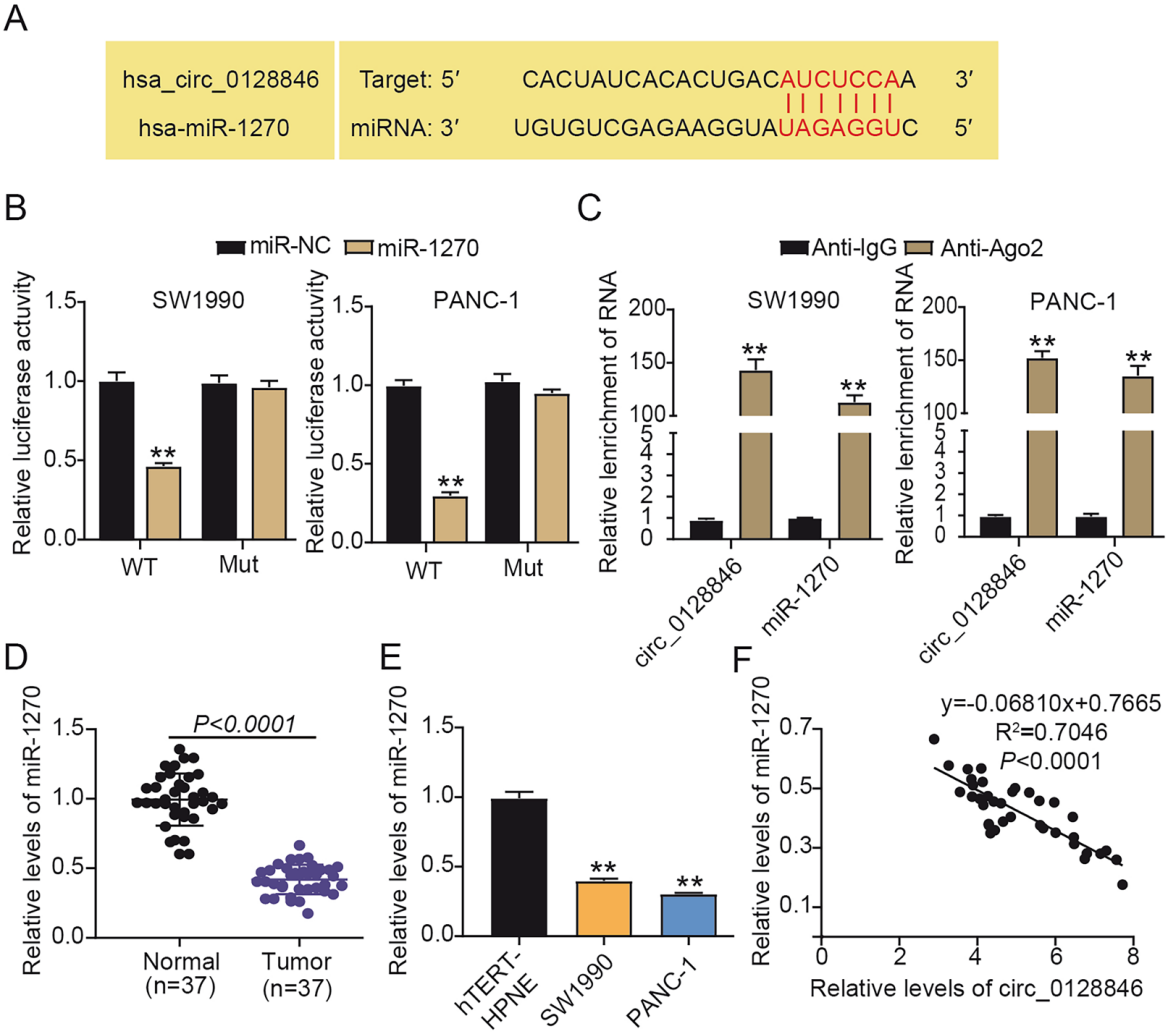
MiR-1270 was a direct target of circ_0128846. (**A**) The binding site between miR-1270 and circ_0128846 was predicted by circinteractome. (**B**) The binding relationship between circ_0128846 and miR-1270 was further testified by dual-luciferase reporter assay. **P < 0.001 relative to miR-NC. (**C**) The binding relationship between circ_0128846 and miR-1270 was vertified by RIP assay. **P < 0.001 relative to Anti-IgG. (**D**) MiR-1270 expression in tumor samples and normal samples was examined by RT-qPCR. (**E**) MiR-1270 expression in PC cell lines (SW1990 and PANC-1) and control cell line (hTERT-HPNE) was examined by RT-qPCR, **P < 0.001 relative to hTERT-HPNE. (**F**) The linear relationship between miR-1270 and circ_0128846 expression in tumor samples was analyzed by Pearson’s correlation analysis.

By the prediction of circinteractome, we noticed that miR-1270 might be one of the targets of circ_0128846 because circ_0128846 contained miR-1270 binding site (Fig. 4A). In addition, miR-1270 accumulation in SW1990 and PANC-1 cells reduced luciferase activity of circ_0128846 WT construct but unchanged luciferase activity of circ_0128846 MUT construct (Fig. 4B). Furthermore, the RNA complexes enriched by anti-Ago2 from SW1990 and PANC-1 cells displayed abundant expression of circ_0128846 and miR-1270 compared to anti-IgG (Fig. 4C). The data verified the binding of circ_0128846 to miR-1270. Next, we demonstrated that miR-1270 expression was considerably declined in pancreatic tumor samples and cell lines (SW1990 and PANC-1) in comparison to normal samples and non-cancer cell line (hTERT-HPNE) (Fig. 4D,E). Besides, miR-1270 expression in tumor samples was notably negatively associated with circ_0128846 expression (Fig. 4F). Overall, miR-1270 was poorly expressed in PC and served as a target of circ_0128846.

### Silencing circ_0128846 repressed PC cell malignant development by upregulating miR-1270

**Figure 5:**
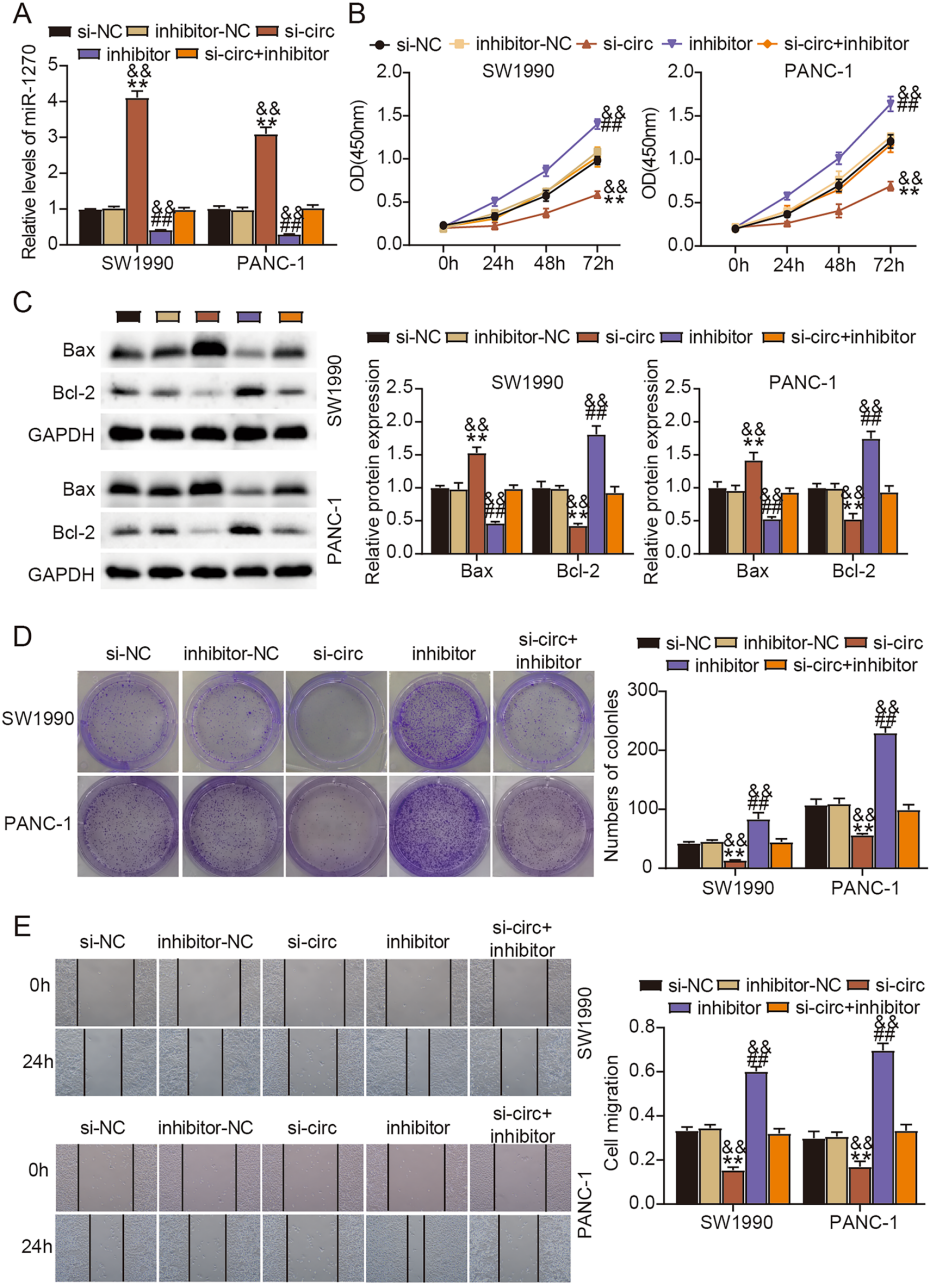
MiR-1270 inhibition reversed the cancer-suppressive effects of silencing circ_0128846 in PC cells. (**A**–**E**) Rescue experiments were conducted in SW1990 and PANC-1 cells with si-NC, inhibitor-NC, si-circ-1, inhibitor or si-circ-1 + inhibitor transfection. (**A**) MiR-1270 expression in these cells was checked by RT-qPCR. (**B**) Cell proliferation was evaluated by CCK-8 assay. (**C**) Expression of Bax and Bcl-2 was measured by western blotting to assess cell apoptosis. (**D**) Cell clone formation ability was evaluated by colony formation assay. (**E**) Cell migration was evaluated by wound healing assay. **P < 0.001 relative to si-NC; ^##^*P* < 0.001 relative to inhibitor-NC; ^&&^*P* < 0.001 relative to si-circ-1 + inhibitor.

We subsequently conducted rescue experiments to determine the interplays between circ_0128846 and miR-1270 on PC cell development. MiR-1270 expression was greatly strengthened in si-circ-transfected cells but greatly reduced in inhibitor-transfected cells; besides, miR-1270 expression was largely repressed by si-circ + inhibitor transfection relative to alone si-circ transfection (Fig. 5A). In function, miR-1270 inhibition enhanced SW1990 and PANC-1 cell proliferation, which was opposite to the effect of silencing circ_0128846; besides, silencing circ_0128846-blocked cell proliferation was largely recovered by further miR-1270 inhibition (Fig. 5B). MiR-1270 absence weakened Bax expression and enhanced Bcl-2 expression, and silencing circ_0128846-induced Bax upregulation and Bcl-2 downregulation were largely reversed by miR-1270 inhibition (Fig. 5C). Additionally, miR-1270 absence also strengthened SW1990 and PANC-1 cell colony-forming and migratory abilities, and its absence partly reversed the role of silencing circ_0128846 to thus recover cell colony-forming and migratory abilities that were suppressed by silencing circ_0128846 (Fig. 5D,E). We concluded from these findings that silencing circ_0128846 repressed PC cell malignant development by upregulating miR-1270.

### NR3C1 was targeted by miR-1270

**Figure 6:**
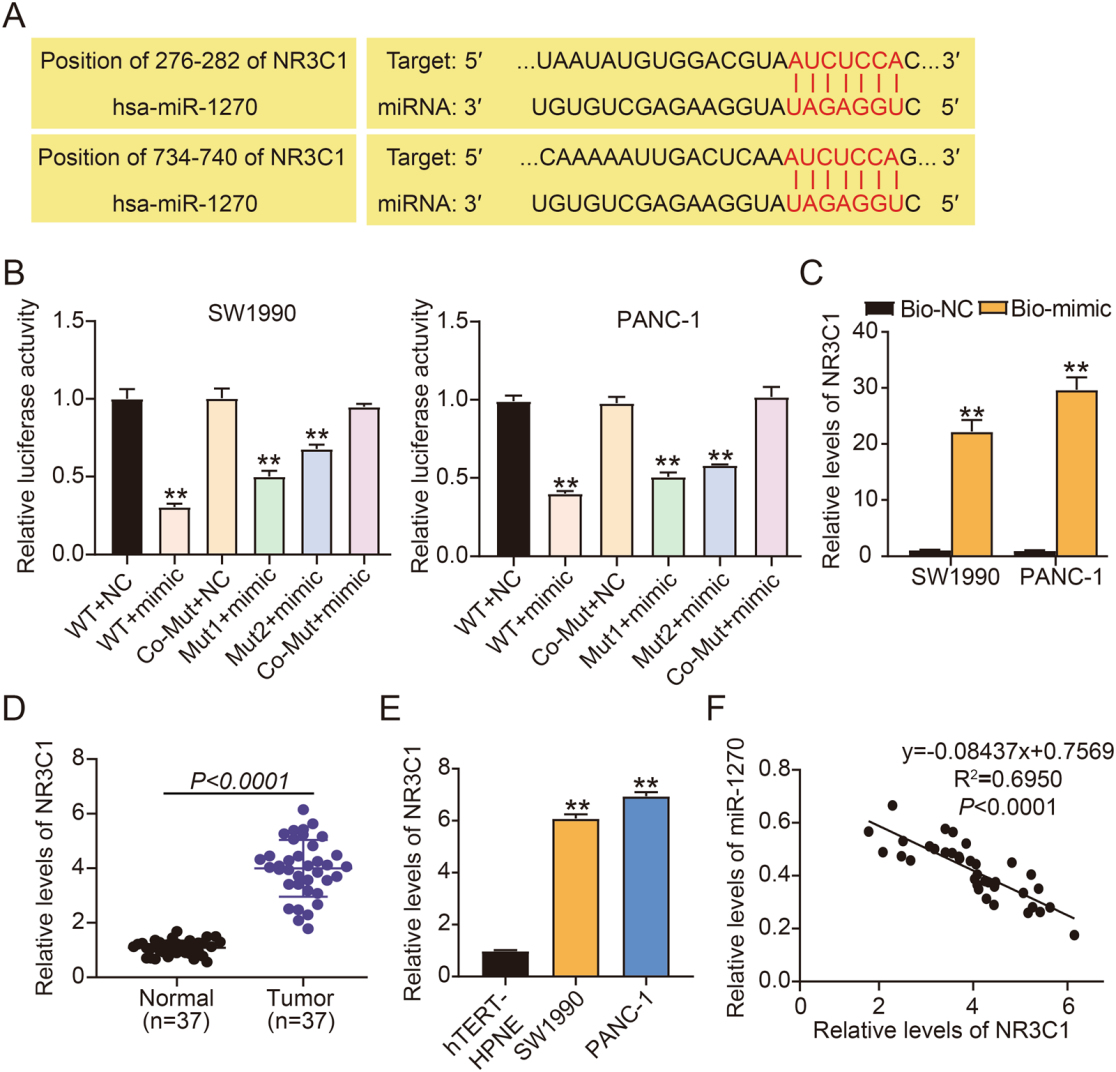
NR3C1 was directly targeted by miR-1270. (**A**) The binding sites between miR-1270 and NR3C1 were predicted by TargetScan. (**B**) The binding relationship between miR-1270 and NR3C1 was further testified by dual-luciferase reporter assay. **P < 0.001 relative to WT + NC. (**C**) The binding relationship between miR-1270 and NR3C1 was verified by pull-down assay. **P < 0.001 relative to Bio-NC. (**D**) NR3C1 expression in tumor samples and normal samples was examined by RT-qPCR. (**E**) NR3C1 expression in PC cell lines (SW1990 and PANC-1) and non-cancer cell line (hTERT-HPNE) was examined by RT-qPCR, **P < 0.001 relative to hTERT-HPNE. (**F**) The association between NR3C1 and miR-1270 expression in tumor samples was analyzed by Pearson’s correlation analysis.

We further investigated the target genes of miR-1270 and obtained from TargetScan that miR-1270 had binding sites with NR3C1 3’UTR at 2 positions (Fig. 6A). Then, we constructed different luciferase reporter plasmids of NR3C1 to testify the predicted binding sites between miR-1270 and NR3C1. The results showed that miR-1270 enrichment largely reduced luciferase activity of NR3C1 WT plasmid and partly reduced luciferase activity of NR3C1 Mut1 or Mut2 plasmid, while miR-1270 enrichment hardly reduced luciferase activity of NR3C1 Co-Mut plasmid (Fig. 6B), verifying that miR-1270 harbored two binding sites with NR3C1. Pull-down assay exposed that high abundance of NR3C1 was enriched by miR-1270 Bio-mimic probe (Fig. 6C), suggesting the binding of miR-1270 to NR3C1. NR3C1 expression was displayed to be highly increased in pancreatic tumor tissues and cell lines (SW1990 and PANC-1), in comparison to normal tissues and normal cell line (hTERT-HPNE) (Fig. 6D,E). MiR-1270 expression in tumor samples was inversely associated with NR3C1 expression (Fig. 6F). The data testified that NR3C1 was targeted by miR-1270.

### The inhibitory effects of NR3C1 knockdown on PC cell malignant phenotypes were attenuated by miR-1270 inhibition

**Figure 7:**
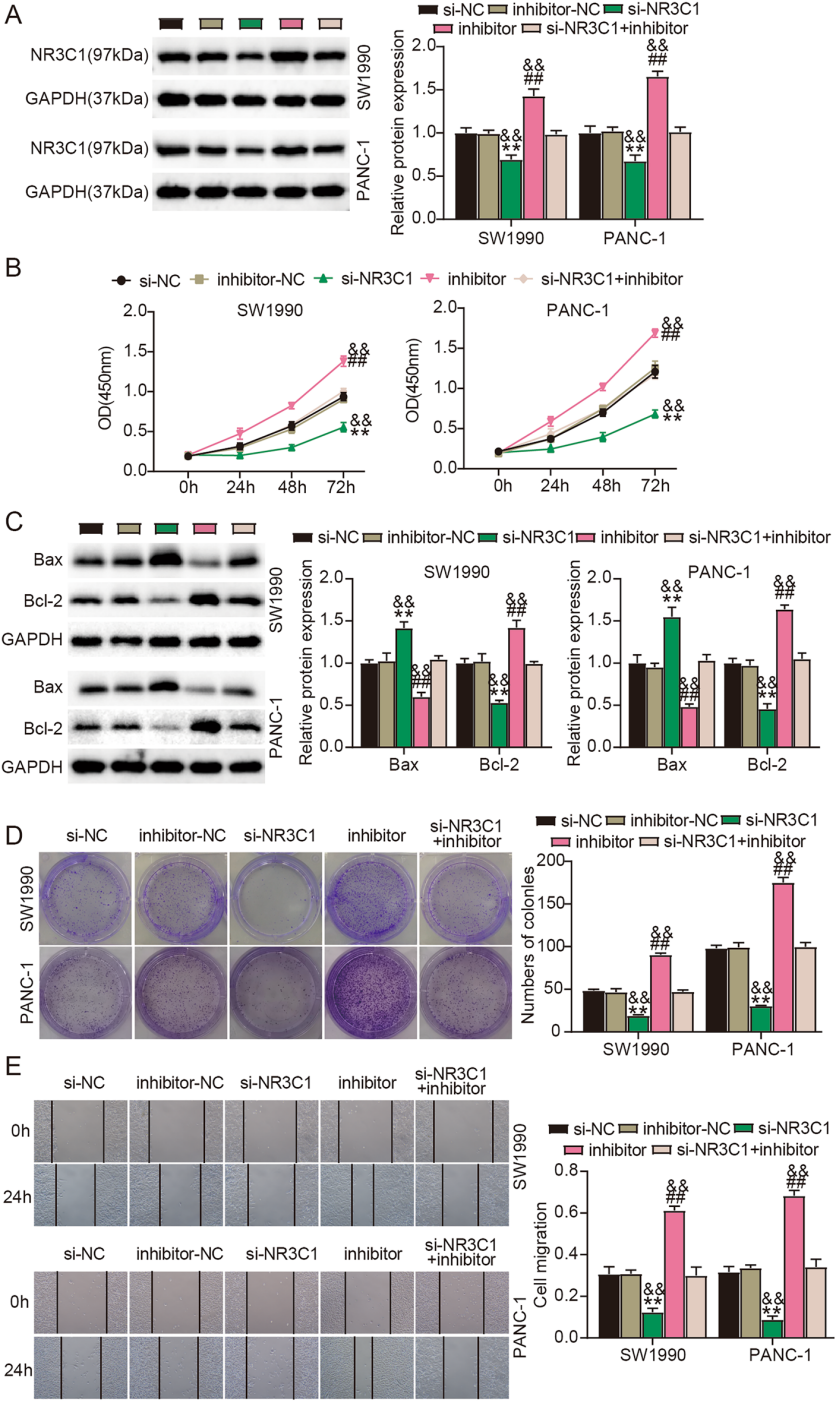
MiR-1270 inhibition reversed the cancer-suppressive effects of NR3C1 knockdown in PC cells. (**A**–**E**) Rescue experiments were conducted in SW1990 and PANC-1 cells with si-NC, inhibitor-NC, si-NR3C1, inhibitor or si- NR3C1 + inhibitor transfection. (**A**) NR3C1 protein expression in these cells was checked by western blotting. (**B**) Cell proliferation was evaluated by CCK-8 assay. (**C**) Expression of Bax and Bcl-2 was measured by western blotting to assess cell apoptosis. (**D**) Cell clone formation ability was evaluated by colony formation assay. (**E**) Cell migration was evaluated by wound healing assay. **P < 0.001 relative to si-NC; ^##^*P* < 0.001 relative to inhibitor-NC; ^&&^*P* < 0.001 relative to si-NR3C1 + inhibitor.

Next, we investigated NR3C1’s function and exploited the interaction between miR-1270 and NR3C1 in PC cell phenotypes. The level of NR3C1 protein was pronouncedly declined in SW1990 and PANC-1 cells with si-NR3C1 transfection but remarkably strengthened in cells with miR-1270 inhibitor transfection; besides, relative to alone si-NR3C1, si-NR3C1 + inhibitor cotransfection partly enhanced the expression of NR3C1 protein (Fig. 7A). In function, NR3C1 knockdown effectively attenuated the proliferation, migratory and colony-forming capacities of SW1990 and PANC-1 cells, while the reintroduction of miR-1270 inhibitor partly abolished the effects caused by NR3C1 knockdown and thus restored cell proliferative, colony-forming and migratory abilities (Fig. 7B,D,E). In addition, we observed that NR3C1 knockdown reinforced Bax expression and impaired Bcl-2 expression in cancer cells, while miR-1270 inhibitor reintroduction attenuated Bax expression that was reinforced by NR3C1 knockdown and recovered Bcl-2 expression that was impaired by NR3C1 knockdown (Fig. 7C). Overall, miR-1270 inhibition reinforced NR3C1 expression and thus attenuated the anti-cancer effects of NR3C1 knockdown on PC cell phenotypes.

## Discussion

Our present study mainly exploited the role of circ_0128846 whose function in PC has never been studied. Our findings discovered that circ_0128846 was overly expressed in PC specimens and cell lines. Silencing circ_0128846 effectively restrained PC cell malignant growth and migration, as well as tumor development in vivo. Moreover, we disclosed the potential functional mechanism of circ_0128846 and revealed that circ_0128846 competitively decoyed miR-1270 to regulate NR3C1 and thus promoted the malignant progression of PC.

Growing evidence has validated the considerable roles of circRNAs in PC, uncovering that circRNAs have great potency to modulate cancer cell growth, migration, invasiveness and multiple phenotypes^21–23^. Regarding to circ_0128846, Wang et al. published that circ_0128846 was greatly overexpressed in colorectal cancer, and circ_0128846 deficiency restrained colorectal cancer cell growth, migratory and invasive abilities; its downregulation in animal models also impeded tumor development^10^. In addition to cancer, circ_0128846 was also recorded to play important parts in osteoarthritis development, showing that circ_0128846 was abundantly expressed in osteoarthritis pathological samples and its downregulation restrained chondrocyte apoptosis, inflammation and cartilage extracellular matrix degradation^24^. These observations unveiled the vital role of circ_0128846 in different human diseases. Interestingly, its aberrant expression elevation in PDAC by microarray analysis was previously recorded in GSE69362 dataset, which aroused our interest. Consistent with its expression pattern in colorectal cancer^25^, our results detected that circ_0128846 expression was noticeably enhanced in PC. Function assays manifested that silencing circ_0128846 effectively restrained PC cell proliferative, colony-forming and migratory abilities but stimulated cell apoptosis. Meanwhile, its knockdown in animal models largely restrained tumor growth. These outcomes indicated that circ_0128846 played cancer-promoting effects in PC.

In view of circ_0128846’s regulatory mechanism, we investigated the miRNAs targeted by circ_0128846 and confirmed the binding of circ_0128846 to miR-1270 by dual-luciferase reporter and RIP experiments. By viewing the previous literature, we found that miR-1270 played contradictory roles in different types of cancer. For example, miR-1270 was highly expressed in osteosarcoma and papillary thyroid cancer, and its high expression was linked to poor prognosis and aggravated cancer cell proliferation and migration^26,27^. In contrast, miR-1270 expression was greatly reduced in glioblastoma and breast cancer, and miR-1270 upregulation effectively blocked the malignant cell behaviors of these cancers^12,13^. Our study characterized the anti-cancer role of miR-1270 in PC, evidenced by its poor expression in PC samples and the promoting effects of miR-1270 inhibition on PC cell proliferation, colony formation and migration. In addition, we discovered that miR-1270 depletion largely reversed the functional effects of silencing circ_0128846, verifying that circ_0128846 bound to miR-1270 to participate in PC progression.

Furthermore, we testified the binding relationship between miR-1270 and NR3C1 by dual-luciferase reporter and pull-down experiments, hinting that NR3C1 might be a target downstream of circ_0128846/miR-1270. NR3C1 expression was viewed to be overexpressed in breast cancer, and the repression of NR3C1 blocked breast cancer cell migration and invasion^20^. In addition to solid tumors, NR3C1 high expression also contributed to the development of hematologic malignancies, such as acute myeloid leukemia^18^. Of note, NR3C1 was greatly downregulated in colon cancer, and miR-19b could suppress NR3C1 expression to strengthen oxaliplatin resistance and colon cancer malignant progression^16^. Our findings discovered that NR3C1 expression was markedly elevated in PC, and NR3C1 knockdown largely repressed PC cell proliferative, colony-forming and migratory abilities but stimulated cell apoptosis. However, these suppressive effects caused by NR3C1 knockdown were partly abolished by miR-1270 inhibition, validating the interactions between miR-1270 and NR3C1 in PC development.

Some limitations still exist in our present work. For instance, the number of clinical specimens is insufficient, and more specimens should be collected to further validate the expressions of circ_0128846, miR-1270 and NR3C1. The relationship of circ_0128846 expression associated with prognosis of patients with PC is unclear and needs to be summarized to clarify. In addition, NR3C1 mutations are associated with glucocorticoid resistance in acute lymphoblastic leukemia^28^. Whether circ_0128846/miR-1270/NR3C1 axis can become a therapeutic target for PC by regulating glucocorticoid resistance to affect cell proliferation, colony-forming and migration needs to further explore. These issues should be focused in future work.

Our study validated the aberrant overexpression of circ_0128846 in PC. We verified the cancer-promoting effects of circ_0128846 in PC, evidenced by the repressive effects of silencing circ_0128846 on cancer cell growth, survival and migration, as well as in vivo tumor development. Circ_0128846 exerted these cancer-promoting effects by targeting the miR-1270/NR3C1 network, at least in part, hinting that the repression of circ_0128846 might be helpful to PC treatment.

## Supplementary Information

Supplementary Information.

## Data Availability

All data generated or analyzed during this study are included in this article.
