# Supplementary material for: Hsa_circ_0128846 knockdown attenuates the progression of pancreatic cancer by targeting miR-1270/NR3C1 axis
Source: Sci Rep. 2023 Feb 16;13:2792. doi: 10.1038/s41598-023-28439-w (PMC9935855; doi:10.1038/s41598-023-28439-w)

PANC-1 Bax for Fig 2C

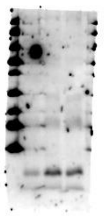

PANC-1 Bcl-2 for Fig 2C

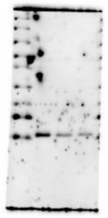

PANC-1 GAPDH for Fig 2C

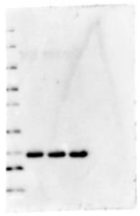

SW1990 Bax for Fig 2C

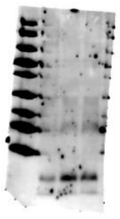

SW1990 Bcl-2 for Fig 2C

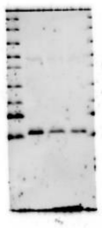

SW1990 GAPDH for Fig 2C

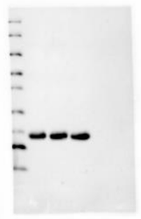

PANC-1 Bax for Fig 5C

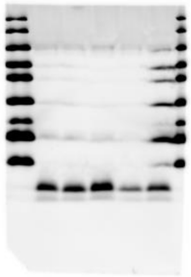

SW1990 Bax for Fig 5C

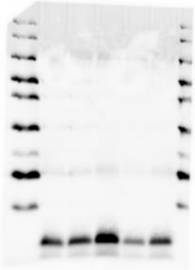

PANC-1 Bcl 2 for Fig 5C

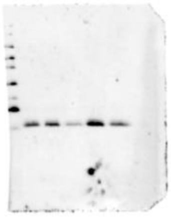

SW1990 Bcl 2 for Fig 5C

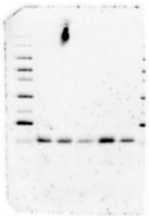

PANC-1 GAPDH for Fig 5C

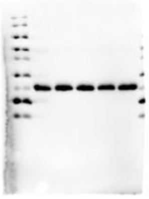

SW1990 GAPDH for Fig 5C

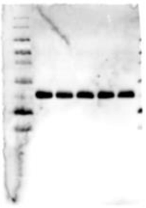

PANC-1 GAPDH for Fig 7A

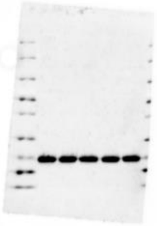

SW1990 GAPDH for Fig 7A

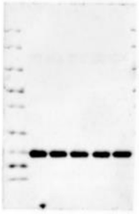

PANC-1 NR3C1 for Fig 7A

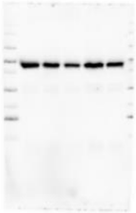

SW1990 NR3C1 for Fig 7A

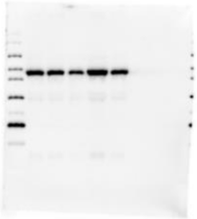

PANC-1 Bax for Fig 7C

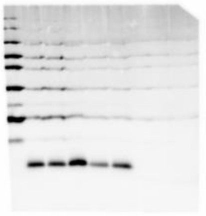

SW1990 Bax for Fig 7C

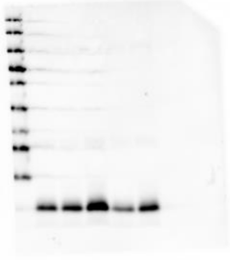

PANC-1 Bcl-2 for Fig 7C

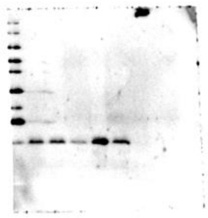

SW1990 Bcl-2 for Fig 7C

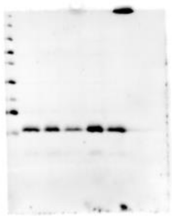

PANC-1 GAPDH for Fig 7C

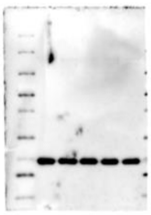

SW1990 GAPDH for Fig 7C

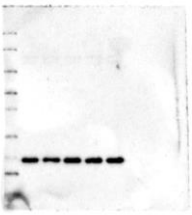

Supplement: Supplementary file 1 — Supplementary Information. [file 41598_2023_28439_MOESM1_ESM.pdf]
